# Dynamic fluorescence lifetime sensing with CMOS single-photon avalanche diode arrays and deep learning processors

**DOI:** 10.1364/BOE.425663

**Published:** 2021-05-17

**Authors:** Dong Xiao, Zhenya Zang, Natakorn Sapermsap, Quan Wang, Wujun Xie, Yu Chen, David Day Uei Li

**Affiliations:** 1 Strathclyde Institute of Pharmacy and Biomedical Sciences, University of Strathclyde, Glasgow G4 0RE, Scotland, UK; 2 Department of Biomedical Engineering, University of Strathclyde, Glasgow G1 1XQ, Scotland, UK; 3 Department of Physics, University of Strathclyde, Glasgow, G4 0RE, Scotland, UK

## Abstract

Measuring fluorescence lifetimes of fast-moving cells or particles have broad applications in biomedical sciences. This paper presents a dynamic fluorescence lifetime sensing (DFLS) system based on the time-correlated single-photon counting (TCSPC) principle. It integrates a CMOS 192 × 128 single-photon avalanche diode (SPAD) array, offering an enormous photon-counting throughput without pile-up effects. We also proposed a quantized convolutional neural network (QCNN) algorithm and designed a field-programmable gate array embedded processor for fluorescence lifetime determinations. The processor uses a simple architecture, showing unparallel advantages in accuracy, analysis speed, and power consumption. It can resolve fluorescence lifetimes against disturbing noise. We evaluated the DFLS system using fluorescence dyes and fluorophore-tagged microspheres. The system can effectively measure fluorescence lifetimes within a single exposure period of the SPAD sensor, paving the way for portable time-resolved devices and shows potential in various applications.

## 1. Introduction

The fluorescence lifetime (FL) indicates the average time a molecule stays excited state before returning to the ground state [1]. Measuring FL can reveal abundant cellular or molecular information, such as cells’ microenvironments (temperature, pH, and ion concentrations [2,3]). It is also affected by non-radiative energy quenching processes (for example, energy coupling between two proteins within 10nm, known as Förster resonance energy transfer or FRET). Thus, FRET-based lifetime measurements provide a means to observe protein interactions or protein conformational changes [4,5]. Furthermore, FL delivers better quantitative analysis because it is less susceptible to fluorochromes’ excitation/emission spectra and concentration variations. It can also distinguish tagged fluorochromes on specified cells from background signals (such as autofluorescence) or unbounded fluorochromes. Fluorescence lifetime imaging microscopy (FLIM) techniques have been widely used in biomedical research, from single-molecule analysis to clinic diagnosis [6]. However, although FLIM instruments have been significantly enhanced, they are still too slow for 3D cellular imaging, endoscopy, and observing fast-moving particles or cells. For applications such as monitoring dynamic changes in a large cell population, high-throughput drug screening, or transient biological dynamics, measuring fast FL variations still poses a significant challenge on current FL-based systems.

There are time- and frequency-domain methods for FL measurements [1]. Previously, frequency-domain methods are predominantly adopted for dynamic FL measurements because they are less complicated and fast. For example, frequency-domain phase-sensitive approaches were demonstrated to measure FLs of flowing cells or particles [7–9]. However, frequency-domain methods show a lower temporal resolution and are less photon-efficient. Time-correlated single-photon counting (TCSPC) is the gold standard time-domain technique due to its superior temporal resolution, photon efficiency, and signal-to-noise ratio (SNR) performances [10]. It is beneficial for live-cell measurements under low light conditions. However, TCSPC is not yet widespread in dynamic FL measurement. Only a few TCSPC-based FL measurement systems have been reported, such as high-throughput TCSPC and time-domain FL flow cytometry systems [11–13]. There are two main challenges for integrating TCSPC in dynamic FL measurement. Firstly, TCSPC suffers from a low photon-counting throughput in traditional single-photon detectors (SPDs). Because of SPDs’ and time-to-digital converters’ dead-times, the photon detection rate in standard TCPSC systems should correctly set (typically below 5% laser repetition rate) to avoid pile-up effects distorting the measured lifetime. Also, traditional SPDs, such as photomultipliers (PMTs) and microchannel plates (MCPs), have only one or several timing channels. Therefore, the photon throughput is usually limited. For the dynamic FL measurement, cells pass through the laser interrogation point with a high velocity, leading to a limited measurement time. Besides, the illumination is kept low to avoid photo-bleaching. The emitted fluorescence signal, therefore, has a low signal-to-noise ratio (SNR). Both factors essentially limit the application of TCSPC in dynamic FL measurement. The latest hybrid PMTs with an ultrashort dead-time TDC show a remarkable short dead time that can improve the photon-counting throughput to some extent. But the limited channel number is still inadequate for high-throughput FL measurement. Secondly, conventional TCPSC systems usually use nonlinear least-square deconvolution or maximum likelihood methods to estimate fluorescence lifetimes from measured fluorescence decays [6]. They are, in general, computationally intensive and time-consuming, only suitable for offline analysis. Recently, real-time center-of-mass [14–17] and phasor methods have been widely applied in various applications [18,19]. However, they are only suitable for high SNR conditions and sensitive to noise. There is still a lack of advanced algorithms for real-time and low SNR situations.

Rapid advances in silicon (CMOS) manufacturing technologies have allowed single-photon avalanche diodes (SPAD) to be implemented in 2D arrays, offering a much higher photon-counting throughput. CMOS SPAD arrays integrate sensing pixels, timing electronics and other digital processing units in a single chip; they are compact, operate at a low voltage and consume much less power than traditional PMTs [20–22]. Ideally, a CMOS SPAD array with *N* pixels can improve the photon counting speed by *N*-fold compared to single-channel SPDs. CMOS SPAD arrays promise for miniaturized TCSPC systems and broader applications. They have been successfully applied in various applications including time-of-flight ranging, LIDAR, FLIM, and optical tomography [23–27]. Emerging deep learning (DL) techniques have brought a step-change for various areas due to the compelling ability to learning from complex data. DL approaches also pave the way for fast lifetime estimations without resorting to complicated fitting routines. Some pioneer studies have proposed fast data-driven DL methods to analyze FLIM images with high accuracy [28,29]. Our previous study proposed a highly efficient DL hardware-friendly architecture for fast and accurate fluorescence lifetime analysis [30]. It is not only suitable for general-purpose processors but also embedded platforms such as field-programmable gate array (FPGA) and application-specific integrated circuit (ASIC). Hardware-friendly DL techniques allow the developments of more cost-effective, portable, and compact DFLS systems without relying on slow analysis software.

We developed a dynamic fluorescence lifetime sensing (DFLS) system with a 192×128-pixel array CMOS SPAD sensor (QuantiCAM) [22] and a DL hardware processor. The DFLS system is a simplified time-resolved flow cytometry system specified for fast FL estimations. QuantiCAM was initially designed for general-purpose LiDAR and FLIM applications with a tunable timing resolution [22]. Our work features two innovations. First, we reconfigured the SPAD array as a megapixel sensor (reading out timestamps of all pixels from a single exposure period, or a single frame, to deliver the fluorescence decay) in our flow cytometry system. The advantages of such a configuration are apparent. Current state-of-the-art CMOS SPAD arrays still suffer from limited sensing area with low fill factors, high dark count rates and low photon detection efficiency. QuantiCAM has a fill factor of 42%, much larger than previously reported sensors, offering better photon collection efficiency [16,23]. Moreover, each pixel has a resolution tunable TDC. All pixels work in parallel, significantly improving the photon throughput. Second, we developed a DL processor based on a quantized convolution neural network (QCNN) for fast lifetime analysis. QCNN is specified for FPGA acceleration, showing high accuracy with an extensive dynamic range and low energy/resource consumptions. The proposed QCNN and the DL processor's performances were quantitatively analyzed, and the whole system was evaluated using fluorescein dye solutions and fluorophore-tagged microspheres.

## 2. System overview

**Fig. 1.**
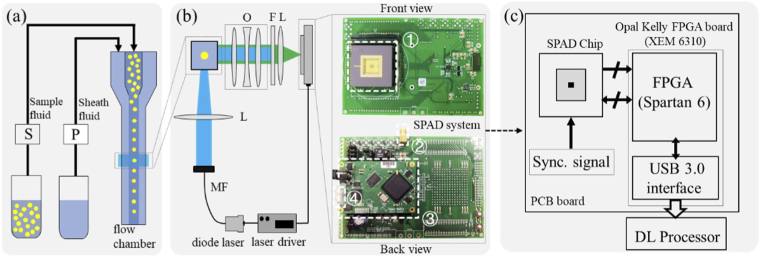
Overview of the DFLS system. (a) The fluidic system. A syringe and a peristaltic pump pumped the sample and sheath flow into the flow chamber, respectively. (b) The optical path and the sensor system. The pulsed diode laser is coupled to a multimode fiber (MF) to collimate the light. The laser is focused by an aspheric lens (*L*) and illuminates the flow chamber. The emitted fluorescence signal is collected at the 90° scattered side after passing through an objective lens (*O*), a long-pass filter (*F*), and an aspheric lens. The inset shows the front and back views of the SPAD system. The main components are labeled in white boxes: ① the sensor chip, ② the interface for synchronized signals from the pulsed laser, ③ the Opal Kelly board, and ④ the USB3 interface. (c) Block diagram of the SPAD sensor system. The raw data are fetched from the SPAD and then processed by the DL processor.

The proposed DFLS system comprises four subsystems: the fluidic system, the optical path, the SPAD system with timing electronics, and the DL processor for FL estimation. Figure 1(a) shows the schematical view of the fluidic system, which delivers cells or particles from a solution to the laser interrogation area. The sample fluid is injected by a 1 ml syringe with an inner diameter of 4.7 mm (Terumo, UK) driven by a syringe pump (NE-300, Jaytee, UK). The sheath flow is pumped by a peristaltic pump (Multiflow, Lambda Instrument, Switzerland), driving the sample flow into a central core, allowing cells/particles to pass the uniformly illuminated area. The flow cytometry cuvette adopts a 250 µm × 250 µm quartz square flow channel (Fireflysci, USA). Both the sample and sheath flow are simultaneously injected into the flow cytometry cuvette by a cross-connector assembly (P-634, Kinesis, Dublin, Ireland), and the end of the cuvette connects to the waste line through a tee-connector assembly (P-701, Kinesis, Ireland). The sample and sheath fluid are recycled by a beaker. The top view of the optical path and the SPAD sensor system are shown in Fig. 1(b). A MicroLED pulsed diode laser (Horiba Scientific, UK) generated picosecond laser pulses with a pulse width < 200 ps. The laser is connected to a multi-core fibre to collimate light and excite flowing samples in the flow chamber. The fluorescence light is collected at the 90° scattered side by an objective lens (40L/0.65NA, Spindler & Hoyer, Germany) and passes through a long-pass filter. An aspheric lens (Thorlabs) focuses on the fluorescence, and the SPAD sensor is placed on the focal plane to detect fluorescent photons.

**Fig. 2.**
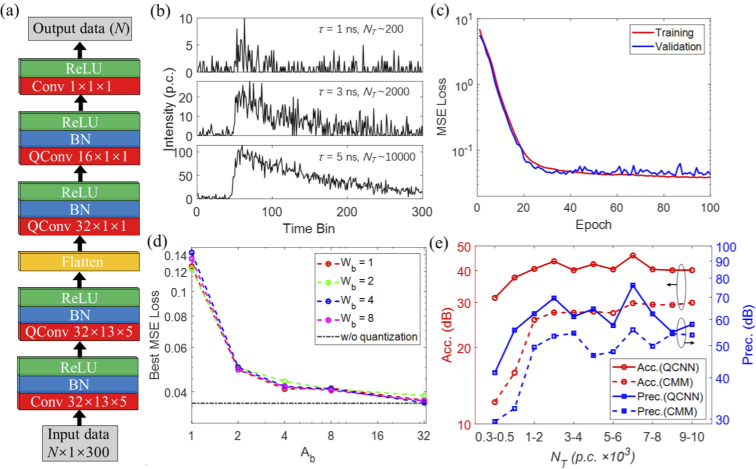
Design and evaluation of the quantized convolutional neural network. (a) The architecture of the neural network. Each block contains a (quantized) convolutional neural network (QConv), a batch normalization (BN) layer, and a rectifier linear unit (ReLU) activation layer. The convolutional layer parameters are the filter number × the kernel size × the stride. (b) Simulated training data with different lifetimes and photon counts. (c) Training and validation of the QCNN with *W_b_* = 1 and *A_b_*_ _= 4. (d) The best MSE loss changes with different weights and activation quantization bits. (e) Accuracy and precision plots of lifetime determinations using QCNN and CMM.

The inset in Fig. 2(b) shows the detailed view of the QuantiCAM camera. The SPAD array was manufactured in STMicroelectronics’ 40-nm CMOS process technology in a 3.15 mm × 2.37 mm chip. A printed circuit board provides an interface for both the SPAD chip and an FPGA board (XEM6310, Opal Kelly). It also provides an input interface for the laser driver's synchronized signal, which is used as a reference signal for TCSPC timing. The FPGA provides wide bandwidth for data transfer and sensor configurations. The detailed design and fabrication of this SPAD chip were reported in [22]. Each pixel has an individual 12-bit time-to-digital converter (TDC) offering 33–120 ps adjustable resolution according to its excess bias (*V*_EB_). The SPAD’s peak detection probability is 34% at 560 nm for *V*_EB_ = 1 V, and the median dark count rate is 25Hz under 1.5V bias. The maximum frame rate and I/O rate of QuantiCAM can reach 18.6k fps and 6.4 Gbps, respectively. We read out timestamps of all pixels of the SPAD array (from a single exposure frame) to deliver a single fluorescence decay. Figure 1(c) shows the block diagram of the embedded system architecture. When a pixel of the SPAD array detects a photon event, a Nuclear Instrumentation Module (NIM) standard signal is generated and delivered to the FPGA chip for post-processing. The Opal Kelly board also provides a USB3 interface for sensor configurations and data transfer. In our experiments, the TDC resolution was fixed at 39 ps, and 300 TDC time bins were used. The frame rate is 1k fps, indicating that the measurement time of 1ms for one frame.

## 3. Deep learning processor for lifetime analysis

We developed a quantized convolutional neural network (QCNN) specified for embedded hardware devices like FPGAs to accurately reconstruct the lifetime from low SNR data with complex noise features. To design a highly efficient neural network with high-throughput, low-latency, and low energy consumption, we adopt the one-dimensional convolutional neural network architecture. The design and evaluation of this architecture were discussed in detail in [30]. This architecture can rapidly resolve multi-exponential fluorescence decay models with high accuracy. Compared with other high dimensional neural networks, it is flexible and straightforward to tackle input data. Since only average lifetimes need to be measured in our system, the network was tailored for the single-exponential decay model by keeping only one output branch. The topological structure of the QCNN is shown in Fig. 2(a), which comprises five one-dimensional convolution layers with corresponding batch normalization (BN) layers and nonlinear rectifier activation layers (ReLU). The first two layers have large kernel sizes and strides to fast capture high-dimensional abstract features of decay functions. The last three layers are pointwise convolution layers for down pooling the information. The three middle convolution layers are quantized to low bit-width weights and activations apart from the first and last convolution layers. Low bit-width quantization is critical for embedded platforms to achieve superior performances in many aspects. Quantizing a neural network can bring enormous benefits while maintaining accuracy. It can increase network throughput by reducing computational complexity and off-chip data transfer. By quantizing the neural network's weights and activations, computationally expensive convolutions are replaced by fast and cost-effective fixed-point arithmetic or bitwise operations, thus significantly improving the calculation speed. Moreover, the parameter size is reduced substantially. It allows the network to use on-chip caching efficiently without relying on slow off-chip memory. The quantization also significantly reduces the consumption of hardware resources and energy.

To quantize the neural network, the weights and activations of layers in the neural network were quantized to an arbitrary bit width. Here the “DoReFa” quantization scheme is applied [31]. The *k*-bit quantization function *Q*(*r*, *k*) for the forward network parameters is expressed as:

(1) $$Q(r,k)=\frac{1}{2^{k}-1}round((2^{k}-1)r),$$

where *round()* is to round the data to the nearest decimal. The input and output of *Q* are confined in [0, 1]. For the *k*-bit weight quantization, *W_k_* is expressed as:

(2) $$W_{k}=2Q\left(\frac{\text{tanh}(W)}{2max(|\text{tanh}(W)|)}+\frac{1}{2},k\right)-1,$$

when *k* = 1, *W_1_* is simplified as *W*_1 _= sign(*W*)×***E***(|*W*|). Because the previous batch normalization and ReLU layers ensure the activations are within the range of [0, 1], the *k*-bit activation is directly obtained by:

(3) $$A_{k}=Q(A,k)$$

Here only the forward propagation is quantized. For the backward gradient propagation, all the quantized layers follow the “straight-through estimator” method during the training phase.

The neural network is a data-driven approach. The resolvable lifetime range and SNR of the QCNN are determined by the training dataset. Besides, obtaining sufficient sample data is also essential for network training. Synthetic fluorescence decays were carefully devised and generated to serve this purpose. In simulations, the number of time bins is *N* = 300 with a bin width *h *= 0.04 ns, similar to the QuantICAM settings. The ground-truth fluorescence decay is expressed as:

(4) $$\text{y}(\text{t})=N_{T}\cdot IRF(t)*e^{-t/\tau }dt,\;\;\;t\;=\;1,\ldots ,N,$$

where *τ* is the fluorescence lifetime ranging from 0.1 to 5 ns with a uniform distribution. The trained lifetime covers a wide range of lifetimes of commonly used fluorophores for biomedical applications. *IRF*(t) is the equivalent instrument response function of the SPAD array (see details in the next section), approximated by a Gaussian function with FWHM = 230 ps with the peak position at the 50^th^ time bin. The asterisk * denotes the convolution operation and the integral $\int IRF(t)*e^{-t/\tau }dt\;=\;1$. Therefore, *N_T_* is the total photon count of the decay, ranging from 100 to 1e4 p.c. The measured decay is the ground-truth decay contaminated by various noise sources. System noise can be generally categorized into Poisson and non-Poisson noise. The former comes from photon detection's discrete nature, whereas the latter has complex origins, including surrounding scattered light, TDC nonlinearity, circuit clock noise, and quantization noise. We used Gaussian noise with zero mean and a standard deviation *σ* randomly ranging from 1 to N_T_/300. It is an empirical setting based on observations of decays obtained from the sensor. Thus, we can describe the measured decays as:

(5) $$Y(t)=y(t)+\sqrt{y(t)}N(0,1)+N(0,\sigma )$$

where N (*µ, σ*) is the Gaussian distribution with mean *µ* and a standard deviation *σ*. Poisson noise is approximated using a normal distribution. Figure 2(b) shows synthetic samples with different lifetimes and photon counts. As for the network training, the QCNN was implemented in Python with the open-source library *Pytorch* [32]. The training dataset contains 50,000 samples and 20% of which were for validation. The batch size of the training samples is 128, and the training epochs are 100. The loss function is the mean square error (MSE), and the optimizer is the Adam algorithm with a learning rate of 1e-4. The MSE losses of training and validation of the QCNN are shown in Fig. 2(c). Here the weight is one-bit, and the activation is four-bit. After 40 epochs, both converged to a plateau with a loss of 0.02. Thanks to the simple and efficient network architecture, the whole training time is less than 3 minutes with the Intel i9 CPU. The effect of the quantization on the network performance is shown in Fig. 2(d). The best MSE loss of the training phase is used as an indicator to evaluate the degradation of the network performance when the parameters are quantized. The bits of weights and activations are denoted as *W_b_* and *A_b_*, respectively. From Fig. 2(d), *W_b_* has little impact on the best MSE loss even it is quantized to 1 bit. However, a smaller *A_b_* will seriously degrade the network's performance when *A_b_* < 4. The results confirm that the one-bit weight and four-bit activation are the optimal configurations for the network design.

A new dataset that is unseen by the network is used to evaluate QCNN’s performance. The sample number, lifetime range, and intensity range are the same as the training dataset. For a better comparison, the lifetimes estimated by QCNN are compared with those calculated by CMM. CMM is the first available hardware algorithm that has been successfully embedded in hardware for fast FLIM analysis. CMM uses the decay’s center-of-mass to determine the lifetime [14] with bias corrections [15]. From the measured fluorescence decay, the lifetime *τ* is calculated as [15,17]:

(6) $$\tau_{CMM}=\Omega \left[\frac{1}{M}\left(\frac{\sum_{i=1}^{M}D_{i}}{N_{T}}+\frac{1}{2}\right)\right]\cdot Mh,$$

where *M* is the number of time-bin in the measurement window, *h* the bin width, *N_T_* the total photon count, and *D_i_* the number of detected photons in the *i^th^* time bin. *Ω* is the look-up table (LUT) for bias corrections when *t*/(*Mh*) < 1/4. *M* starts from the 50^th^ time bin (the peak position) to the 300^th^ time bin in our calculation. The IRF was considered in lifetime estimations with *τ_CMM_calibrated = _τ_CMM_* - *τ_CMM_IRF_* [17]. To assess the effectiveness of an analysis method, here we defined the accuracy (Acc.) and precision (Prec.) as:

(7) $$Acc.\;(dB)\;=\;20log_{10}\left(\frac{\tau }{\Delta \tau }\right),$$

(8) $$Prec.\;(dB)=\;20log_{10}\left(\frac{\tau }{\sigma \tau }\right),$$

where *Δτ* and *στ* are the absolute error and the standard deviation, respectively. Figure 2(e) shows the mean accuracy and precision of calculated lifetimes versus *N_T_*. QCNN outperforms CMM in accuracy and precision, especially for low count conditions (*N_T_* < 500). The accuracy of QCNN for *N_T_* from 300 to 500 is even better than that of CMM for *N_T_* from 9000 to 10,000. Additionally, the precision of QCNN also outperforms CMM by a large margin. Although CMM is simple, it is sensitive to noise. It is also nonlinear and can deliver misleading estimations when there are more than two lifetime species in the field of view [33]. Instead, QCNN can circumvent manual settings and directly provide better analysis. It is robust and accurate for noisy and low count conditions, suitable for various potential applications.

**Fig. 3.**
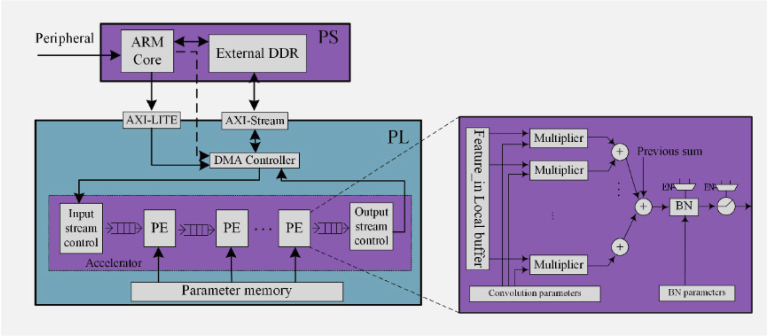
Hardware block diagram for the quantized convolution neural network. The FPGA device can be divided into two parts, the programmable logic (PL) and the processing system (PS), which correspond to the programmable logic circuit and Arm cortex-9 CPU cores. The insert shows the details of the processing element (PE).

The trained QCNN was implemented on an FPGA as a lifetime processor. The FPGA is an ideal platform for prototyping because of its reconfigurability and high-level implementation tools, allowing fast verifications of the proposed neural networks. Figure 3 shows the overall hardware block diagram. The network architecture was designed using the Xilinx Vivado High-Level-Synthesis (HLS) tool. As there are limited digital resources in the Opal Kelly XEM6310 board (with a Xilinx Spartan-6 FPGA), we implemented the DL processor in another FPGA device, ZYNQ 7020 (Xilinx, USA), for this proof-of-concept study. The FPGA device contains programmable logic blocks and dual Arm cortex-9 CPU cores. The former is for establishing the network backbone, whereas the latter is for configuring status registers of the Direct-Memory-Access (DMA) controller and peripherals. The AXI-Stream and AXI-LITE buses are for data transfer and configurations of convolution scale parameters, respectively. Once the model is well trained, all the parameters are parsed and extracted from the model and then initialized in dual-port Block Random-Access Memory (BRAM) modules in the FPGA with processing elements (PE) fetching corresponding parameters for convolution and BN operations. Instead of computing all the input feature data, each PE contains a local buffer that caches a specific length of data to conduct one-time convolution to improve the parallelism. Therefore, all processing elements can execute corresponding operations simultaneously. Additionally, two multiplexers are utilized to configure BN and ReLU modules when they are activated.

**Fig. 4.**
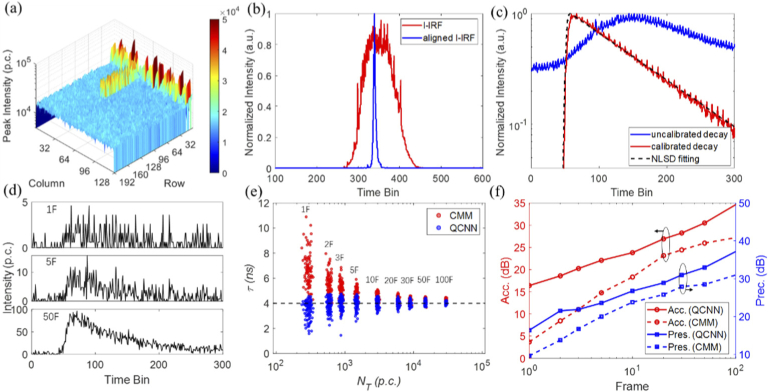
System calibrations and evaluations. (a) The IRF peak intensity distribution. (b) The IRF integrated the whole sensor (I-IRF) (in red) and aligned I-IRF (in blue). (c) The fluorescence decays of fluorescein before and after calibration. The dashed line is the fitting curve using NLSD. (d) The measured decays under different frames. (e) The lifetime distributions calculated by QCNN and CMM as a function of the total photon count, *N_T_*. The frame number has a linear relationship with *N_T_*. (f) The comparison between QCNN and CMM in terms of accuracy and precision.

Table 1 summarizes the main characteristic parameters of the DL lifetime processor. The calculation throughput is up to 54 µs/sample, which is comparable with a mainstream GPU. As a comparison, the calculation throughput of the Intel i7 4790 is around 55 µs/sample. For a single sample, the total number of floating-point operations (FLOPs) is only 0.2 million, showing the high efficiency of the network architecture. The fast training time allows the processor to be rapidly deployed for different lifetime and SNR ranges, which is beneficial for broad applications. The DL processor also has compelling advantages in structure simplicity, throughput, and power consumption. It paves the way for portable and compact fluorescence lifetime sensing devices without resorting to slow software tools.

**Table 1.** Main characteristics of DL lifetime processor.

Total parameters	26,881
FLOPs [M/sample]	0.2
Training time [Min]	3
On-chip memory [Mb]	3.37
Throughput [µs/sample]	54
On-chip power [W]	4.5

## 4. Experimental results

The instrument response function (IRF) of the DFLS system was firstly calibrated. IRF characterizes the system’s overall timing precision. Narrower IRF indicates higher timing resolution. A solution of Ludox was placed at the laser interrogating point to replace the flow chamber to obtain the IRF. The scattered light from the Ludox solution with an ultra-short decay was used as the excitation signal. The long-pass filter was removed to allow the scattered light to pass through, and a neutral density (ND) filter was added in front of the sensor to reduce the light intensity. Figure 4(a) shows the SPAD array's IRF peak intensity distribution; 10^4^ frames in total were recorded. Except for the T-shape area that shows abnormal high peak intensities due to manufacturing and readout defects, the sensor array's intensity profile has a relatively flat distribution, indicating that the optical path was well-positioned. Some pixels showing a high dark count rate (usually called hot pixels) exist randomly across the SPAD array. Figure 4(b) shows the integrated IRF (I-IRF) when the sensor array functions as a single megapixel. Due to manufacturing uncertainties, each pixel and its corresponding TDC behave slightly differently in terms of the response time (related to IRF), noise, jitter, and TDC gain & linearity. Therefore, as indicated by the I-IRF, the sensor array shows an irregular IRF with a significant FWHM over 4 ns. Using such an uncalibrated IRF would severely distort measured decays and lead to incorrect lifetime estimations. To address this problem, pixels need to be aligned according to their peak positions, with hot pixels masked out. The aligned I-IRF has a sharp peak with an FWHM of 0.23 ns, comparable with mainstream FLIM systems. The intensity peak positions and hot pixels are recorded in the FPGA for rapid calibrations.

The photon-counting throughput and lifetime calculations were also evaluated with aqueous fluorescein solutions. Fluorescein shows single-exponential decay characteristics with an excellent quantum yield. It is easy to prepare an ideal fluorophore for flow cytometry characterizations. The fluorescein solution was made by mixing fluorescein powder with water until the solution was saturated before injecting into the flow chamber. The maximum photon-counting throughput reached 1.9×10^4^ counts per frame (cpf) in the experiments. Since we conducted benchmark experiments using fluorophores with known lifetimes, it was easy to ensure that no pile-up effects (fluorescence decay histogram skews towards shorter lifetime) were triggered. Hence the fluorescence lifetime can be accurately determined within only one frame. To mimic low-light conditions and investigate the DL processor's performance under low photon counts, an ND filter was inserted to reduce the intensity to around 300 cpf. Figure 4(c) shows the fluorescence decay with 10^4^ measured frames before and after calibration. The uncalibrated IRF shows considerable background noise and is significantly distorted. As a comparison, after aligning pixel IRFs, removing hot pixels, and removing background noise, the measured decay shows a desirable single-exponential decay. The mean photon count in the first 50 TDC time bins is for estimating background noise. It is worth noting that even the measured decay is well-calibrated, it still has apparent periodic noise. It is from the TDC nonlinearity and clock noise of sensor electronics [34]. Nevertheless, it has little impact on lifetime estimations. As depicted by the dashed line, the measured decay can be well fitted by the nonlinear least-squares deconvolution (NLSD) algorithm. The estimated lifetime is 4.01 ns, in good agreement with the literature [35].

The decays under fewer measured frames are shown in Fig. 4(d). Unlike high count conditions, noise from electronics and background scattered light has no periodic distributions. Hence, it can be approximately described as Gaussian noise. The calculated lifetimes of the measured decays are compared using CMM and QCNN. The results are summarized in Figs. 4(e) and 4(f). The fluorescein solution was measured by integrating 1 to 100 frames, and each measurement was repeated 100 times. The measurement window for CMM is from the peak position (50^th^ time bin) to the last time bin. The lifetime versus the total photon count (*N_T_*) is shown in Fig. 4(e). QCNN outperforms CMM significantly under low-count conditions. Interestingly, CMM delivers a more significant value than the ground truth, whereas QCNN stays closer to the ground truth. One distinct drawback of CMM is its high sensitivity to noise. It is why, under low-count conditions, CMM delivers more deviated results. On the contrary, QCNN has no such problem and is more robust against complex noise. Figure 4(f) shows quantitative lifetime analysis in terms of accuracy and precision defined in Eqs. (7) and (8). QCNN delivers better precision and accuracy than CMM, especially for low photon counts.

The developed DFLS system and DL processor were further tested and characterized using fluorophore-tagged microspheres. Two different samples were used in our experiment, one is crimson fluorescent microspheres (F8831, FluoSphere Polystyrene Microspheres, Thermo Fisher, UK), and another is yellow-green (YG) fluorescent microspheres (F8836, FluoSpheres Polystyrene Microspheres, Thermo Fisher, UK). Both microspheres have a 10 µm average size and were dissolved in an aqueous solution with a concentration of 3.6×10^5^ beads/ml. Before pumping into the flow chamber, the two steady sample solutions’ fluorescence lifetimes were measured using a commercial PMT system (FluoroCube Extreme, Horiba Scientific, UK) as a reference. The two samples show a multi-exponential decay feature, and their lifetimes were calculated by commercial software for multiexponential decay fitting (DAS6, Horiba Scientific, UK). The average lifetimes for crimson and YG microspheres were *τ*_Crimson_ = 2.1 and *τ*_YG_ = 3.0 ns, respectively. The two sample solutions were separately pumped into the flow chamber at a speed of 0.12 ml/min. For crimson microspheres, the excitation source was a diode laser with a 640 nm peak wavelength, 10 MHz repetition rate and 300mW peak power (DD-635L, Horiba Scientific, UK). A 670 nm long-pass filter was added in front of the SPAD sensor to remove the excitation light. For yellow-green microspheres, the excitation source was changed to a diode laser with a 503 nm peak wavelength, 10 MHz repetition rate and 100mW peak power (DD-510L, Horiba Scientific, UK) and the filter was replaced by a 530 nm long-pass filter.

**Fig. 5.**
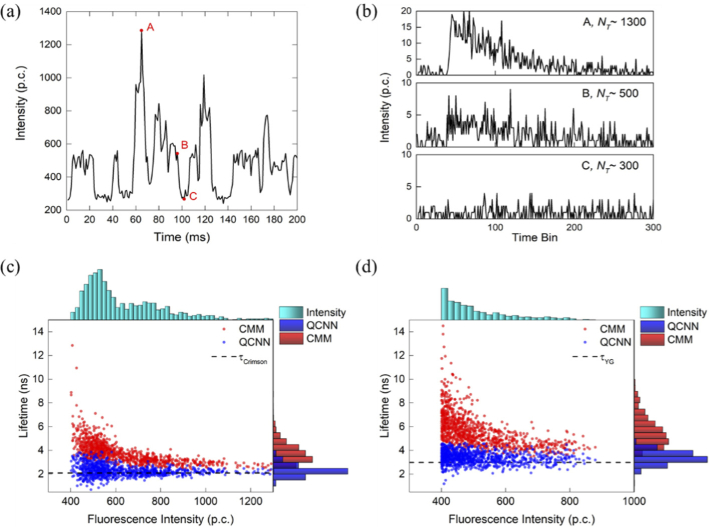
Experimental results of flowing fluorophore-tagged microspheres. (a) The photon detection counts of 200 subsequent frames. Each frame has 1 ms exposure time. (b) The decay histograms of three different frames denoted in (a). (c) and (d), 2D scatter plots of the fluorescence lifetime versus calculated lifetime using CMM and QCNN for crimson and yellow-green tagged microspheres. The upper- and right-side normalized histograms show the distributions of fluorescence intensity and lifetimes, respectively. The bin widths of the intensity and lifetime histograms are 20 p.c. and 0.5 ns, respectively.

To measure the fluorescence lifetime of microsphere events in a flow chamber, one principle is the burst integrated fluorescence lifetime (BIFL) analysis method, in which a long exposure time is set, or sequential frames are segmented into different groups to identify events according to a pre-defined fluorescence intensity threshold [12,13]. However, instead of using BIFL, we analyzed TCSPC data in a single frame with a short exposure time, 1ms per frame, to better evaluate different lifetime estimation algorithms. Figure 5(a) shows a segment of recorded fluorescence intensity of crimson microspheres for 200 ms. Since a different number of microspheres passed through the laser interrogation area within a frame, the detected fluorescence photons per frame have a significant variation ranging from about 300 to 1300 p.c. Figure 5(b) shows fluorescence histograms corresponding to labeled positions *A*, *B*, and *C* on the intensity curve in Fig. 5(a). The measured fluorescence decays are severely contaminated by noise. When no microsphere events are detected, the background noise level is about 300 p.c., mainly contributed by the SPAD dark count. Therefore, an intensity threshold of 400 p.c. is applied to determine detected microsphere events for a frame. It is worth noting that the measured decays of microspheres are similar to the simulated decays shown in Fig. 2(b), indicating that using simulated data is an effective way for network training. Figures 5(c) and 5(d) show 2D scatter plots for the fluorescence intensity versus calculated lifetime using CMM and QCNN. The upper- and right- sides show normalized histograms for measured fluorescence intensities and lifetimes. The fluorescence intensities of YG microspheres are weaker than those of crimson microspheres. It is due to a lower laser power level for YG microspheres. The scatter plots for both samples show similar distributions. Consistent with previous results, CMM delivers significantly biased results, especially for low photon counts. In contrast, the mean lifetimes calculated by QCNN for both samples are close to the reference lifetimes. Table 2 summarises the results calculated by CMM and QCNN. The bias is defined as (*τ_Mean_* - *τ_REF_*)/*τ_REF_*, where *τ_Mean_* is the mean lifetime calculated by CMM or QCNN and *τ_REF_* is the reference lifetime of microspheres. QCNN outperforms CMM in terms of *τ_Mean_*, standard deviation (std), and bias by a significant margin. Results show that our DL processor is more suitable for processing fluorescence signals with a low SNR.

**Table 2.** Fluorescence lifetimes of fluorophore-tagged microspheres

fluorophore	*τ_REF_* (ns)	CMM			QCNN		
		*τ_Mean_* (ns)	*Std*	*Bias*	*τ_Mean_* (ns)	*Std*	*Bias*
Crimson	2.1	3.82	0.94	81.9%	2.3	0.46	9.52%
Yellow-Green	3.0	5.71	1.59	90.3%	3.4	0.50	13.3%

## 5. Discussion

Experimental results show the advantages of the proposed DFLS systems. Owing to the SPAD array sensor with in-pixel TDCs, an enormous photon-counting throughput is achieved. The fluorescence lifetime can be robustly estimated quickly by sacrificing the investigated cells or particles’ spatial information. This is beneficial to applications such as monitoring dynamic behaviours of a large cell population. In our experiments, the exposure time is 1ms per frame. However, the maximum frame rate of the SPAD array sensor can be 18.6 kframes/s. There is still room to improve the system throughput. The DL processor also shows a great capacity to perform lifetime analysis on low-count and noise-contaminated data. Although only the single-exponential decay was considered in this work, our DL architecture can be extended to multi-exponential decay models by adding more output branches as described in [30]. It can be further applied in FRET analysis in flowing cells or imaging applications. The SPAD camera firmware and the DL processor were implemented in two different FPGA boards for a fast proof-of-concept study. Additionally, another noticeable point for the SPAD array sensor is the vast data throughput. As mentioned above, the data transfer rate of our QuantICAM could reach up to 6.4 Gbps. The data transfer in the current firmware still limits the sensor's performance. Therefore, our DFLS system currently cannot conduct an on-the-fly analysis with low latency. Future developments are required to merge modules accommodating the SPAD array sensor and the DL processor in the same FPGA chip for real-time analysis.

## 6. Conclusion

We present a proof-of-concept DFLS system with the QuantiCAM camera and deep learning processors. The 192×128 SPAD array offers a high TCSPC photon-counting throughput. Meanwhile, the DL processor based on quantized convolutional neural networks shows a high calculation throughput with simple architecture, low on-chip memory and power consumption. It can resolve fluorescence lifetimes from low SNR data, and it significantly outperforms the previously reported hardware algorithm, CMM. The whole system was calibrated and tested with fluorescein dye solutions. Flowing fluorophore-tagged microspheres were also characterized. Their fluorescence lifetimes can be accurately evaluated with only one frame. Our DFLS system can be further optimized and shows great potential in various high-throughput and real-time applications.

## Data Availability

The raw data supporting this article's conclusions will be made available by the authors without undue reservation.
